# Genetic variability of porcine pegivirus in pigs from Europe and China and insights into tissue tropism

**DOI:** 10.1038/s41598-019-44642-0

**Published:** 2019-06-03

**Authors:** Johanna Kennedy, Vanessa Maria Pfankuche, Doris Hoeltig, Alexander Postel, Oliver Keuling, Malgorzata Ciurkiewicz, Wolfgang Baumgärtner, Paul Becher, Christine Baechlein

**Affiliations:** 1 Institute of Virology, University of Veterinary Medicine Hannover, 30559 Hannover, Germany; 2 Department of Pathology, University of Veterinary Medicine Hannover, 30559 Hannover, Germany; 3 Clinic for Swine, Small Ruminants, Forensic Medicine and Ambulatory Services, University of Veterinary Medicine Hannover, 30173 Hannover, Germany; 4 Institute for Terrestrial and Aquatic Wildlife Research, University of Veterinary Medicine Hannover, 30173 Hannover, Germany; 5 German Center for Infection Research (DZIF), Partner Site Hannover-Braunschweig, 30559 Hannover, Germany

**Keywords:** Viral evolution, Viral infection

## Abstract

Pegiviruses belong to the family *Flaviviridae* and have been found in humans and other mammalian species. To date eleven different pegivirus species (*Pegivirus A-K*) have been described. However, little is known about the tissue tropism and replication of pegiviruses. In 2016, a so far unknown porcine pegivirus (PPgV, *Pegivirus K*) was described and persistent infection in the host, similar to human pegivirus, was reported. In this study, qRT-PCR, phylogenetic analyses and fluorescence *in situ* hybridization (FISH) were implemented to detect and quantify PPgV genome content in serum samples from domestic pigs from Europe and Asia, in tissue and peripheral blood mononuclear cell (PBMC) samples and wild boar serum samples from Germany. PPgV was detectable in 2.7% of investigated domestic pigs from Europe and China (viral genome load 2.4 × 10^2^ to 2.0 × 10^6^ PPgV copies/ml), while all wild boar samples were tested negative. Phylogenetic analyses revealed pairwise nucleotide identities >90% among PPgVs. Finally, PPgV was detected in liver, thymus and PBMCs by qRT-PCR and FISH, suggesting liver- and lymphotropism. Taken together, this study provides first insights into the tissue tropism of PPgV and shows its distribution and genetic variability in Europe and China.

## Introduction

Pegiviruses comprise a group of positive-sense, single-stranded RNA viruses, with a genome size of 9–13 kb, that were recently classified into eleven species (*Pegivirus A-K*) within the genus *Pegivirus* in the *Flaviviridae* family^1^. They can infect humans as well as a range of mammalian species, including primates, bats, rodents, horses and pigs^2–9^. While pegiviruses are known to cause persistent infections in humans and horses, their pathogenicity remains largely unknown^1,4,10–12^. Though a pegivirus was identified in horses with Theiler’s Disease in the USA^5^, recent studies imply that viruses of the copiparvovirus group are associated with serum hepatitis in horses^13,14^. Human pegiviruses (HPgV) are distributed globally and viral RNA is present in roughly 750 million people, making it one of the most prevalent human RNA viruses^15^.

Though HPgV was initially thought to be hepatotropic and a possible agent of Non-A-E hepatitis, evidence of viral replication in the liver of infected patients is missing or inconclusive^16–18^. Rather, as HPgV replication has been shown in peripheral blood mononuclear cells (PBMCs) *ex vivo* for several weeks, the virus appears to be lymphotropic^19–21^. Additionally, HPgV RNA has been found in serum microvesicles, which have successfully delivered viral RNA to uninfected PBMCs that then supported HPgV replication *ex vivo*^22^. Interestingly, pegivirus infection in humans may have a beneficial effect on the outcome of human immunodeficiency virus type 1 (HIV-1) infections in individuals co-infected with both viruses, including reduced retroviral loads, slower progression to AIDS and improved survival rates. These benefits are attributed to immune-modulating effects as well as direct and indirect antagonistic mechanisms of HPgV on HIV-1 infection^23^.

Porcine pegiviruses (PPgV) were first described in domestic pigs from Germany in 2016^9^. The study reported a PPgV detection rate of 2.2% (10 of 455) in porcine serum samples and described persistent infection for up to 22 months in three pigs that did not display any clinical signs of disease. Apart from Germany, presence of PPgV has been investigated in North America, where a recent study revealed a PPgV detection rate of 15.1% (24 of 159 samples) in the USA^24^. Additionally, a recent study investigated 469 porcine serum samples from China, 34 (7.25%) of which were found PPgV positive. Samples originated from different age groups and proved an ascending trend in the PPgV positive rate from suckling piglets (1.61%) and nursing piglets (1.85%) to finishing pigs (6.56%) and sows (11.34%)^25^.

In this study we analyzed the presence of PPgV genome in pigs from Europe and Asia. To clarify whether wild boar might play a role in the epidemiology of PPgV, as seen in infections with, for example, classical swine fever virus^26,27^, African swine fever virus^28^ and atypical porcine pestivirus (APPV)^29^, we also investigated the presence of PPgV genome in wild boar serum samples from Germany. To date the primary permissive cell type(s) of HPgV and other pegiviruses remain unknown. For this reason, we analyzed the tissue and cell tropism of PPgV through detection and quantification of viral RNA in tissues and PBMCs from PPgV positive pigs using qRT-PCR and fluorescence *in situ* hybridization (FISH).

## Results

### PPgV RNA in serum samples from Europe and Asia

The *in vitro* transcribed RNA copy standard evidenced a highly efficient qRT-PCR assay that was able to detect ten viral genome copies per reaction at Cq values around 36. PPgV genome was detectable in 47 of 1,736 (2.7%) serum samples from domestic pigs corresponding to 20 out of 132 herds (15.2%) (Table 1). Highest detection rates were found in individual animals from Great Britain (10.3%) and in herds from China (58.3%). In the different age groups investigated here, the PPgV positive rates were 1.9% in animals under 4 weeks of age, 1.2% in fattening pigs over 4 weeks of age, 3.4% in sows and boars, and 10.1% in pigs of unknown age and use (Table 2). Viral loads varied between 2.4 × 10^2^ and 2.0 × 10^6^ PPgV RNA copies/ml serum, with an overall average of 3.8 × 10^5^ copies/ml. For individual countries on average, lowest genome loads were detected in Poland (1.9 × 10^4^ copies/ml) and highest in Italy (7.1 × 10^5^ copies/ml). All 800 wild boar samples were negative for PPgV RNA.

**Table 1:** Porcine pegivirus genome detection rates and viral genome load in serum samples from individual animals and herds from different countries in Europe and Asia^1^.

Country of origin	No. animals	No. herds	No. PPgV pos. animals (%)	No. PPgV pos. herds (%)	Genome load range (copies/ml)	Genome load average (copies/ml)
Germany	652	39	12 (1.8)	8 (20.5)	2.0 × 10^3^–2.0 × 10^6^	3.8 × 10^5^
Poland	186	12	4 (2.2)	3 (25.0)	7.1 × 10^3^–3.3 × 10^4^	1.9 × 10^4^
Italy	200	20	5 (2.5)	2 (10.0)	3.6 × 10^2^–1.6 × 10^6^	5.7 × 10^5^
Serbia	73	7	0 (0)	0 (0)	—	—
Switzerland	120	20	0 (0)	0 (0)	—	—
Great Britain	87	unknown	9 (10.3)	unknown	1.7 × 10^3^–1.2 × 10^6^	5.5 × 10^5^
China	218	12	17 (7.8)	7 (58.3)	2.4 × 10^2^–1.2 × 10^6^	2.9 × 10^5^
Taiwan	200	22	0 (0)	0(0)	—	—
Total	1,736	132	47 (2.7)	20 (15.2)	2.4 × 10^2^–2.0 × 10^6^	3.8 × 10^5^

^1^PPgV, porcine pegivirus; pos., positive.

**Table 2:** Number of pegivirus positive pigs of different age groups from Europe and China.

Country of origin	Sampling year(s)	Piglets <4 weeks old (%)	Fattening pigs >4 weeks old (%)	Sows & boar (%)	Unknown age (%)	Total
Germany	2015–2018	—	6/458 (1.3)	6/166 (3.6)	0/28 (0)	12/652 (1.8)
Poland	2017	1/12 (8.3)	1/82 (1.2)	2/78 (2.6)	0/14 (0)	4/186 (2.2)
Italy	2015	—	4/100 (4.0)	1/100 (1.0)	—	5/200 (2.5)
Serbia	2015	—	0/63 (0)	0/10 (0)	—	0/73 (0)
Switzerland	2015	—	0/120 (0)	—	—	0/120 (0)
Great Britain	2016	—	—	—	9/87 (10.3)	9/87 (10.3)
China	2014	1/96 (1.0)	—	10/103 (9.7)	6/19 (31.6)	17/218 (7.8)
Taiwan	2015	—	0/100 (0)	0/100 (0)	—	0/200 (0)
Total	2014–2018	2/108 (1.9)	11/923 (1.2)	19/557 (3.4)	15/148 (10.1)	47/1,736 (2.7)

### Phylogenetic analyses

Altogether 31 PPgV partial NS3 sequences were obtained from domestic pigs, of which nine were identical to one or more other sequences. In total, ten sequences from Germany, three sequences from Italy, four sequences from Poland, nine sequences from Great Britain and five sequences from China were acquired. Sequences GER/SA/13, GER/SA/91, and PL/159 were identical to one additional sequence each, while IT/77, GB/16, and GB/23 were identical to two further sequences each. In Germany, Poland, and Italy, all identical sequences originated from samples from the same farms, while herd affiliation was unknown for samples from Great Britain.

**Figure 1:**
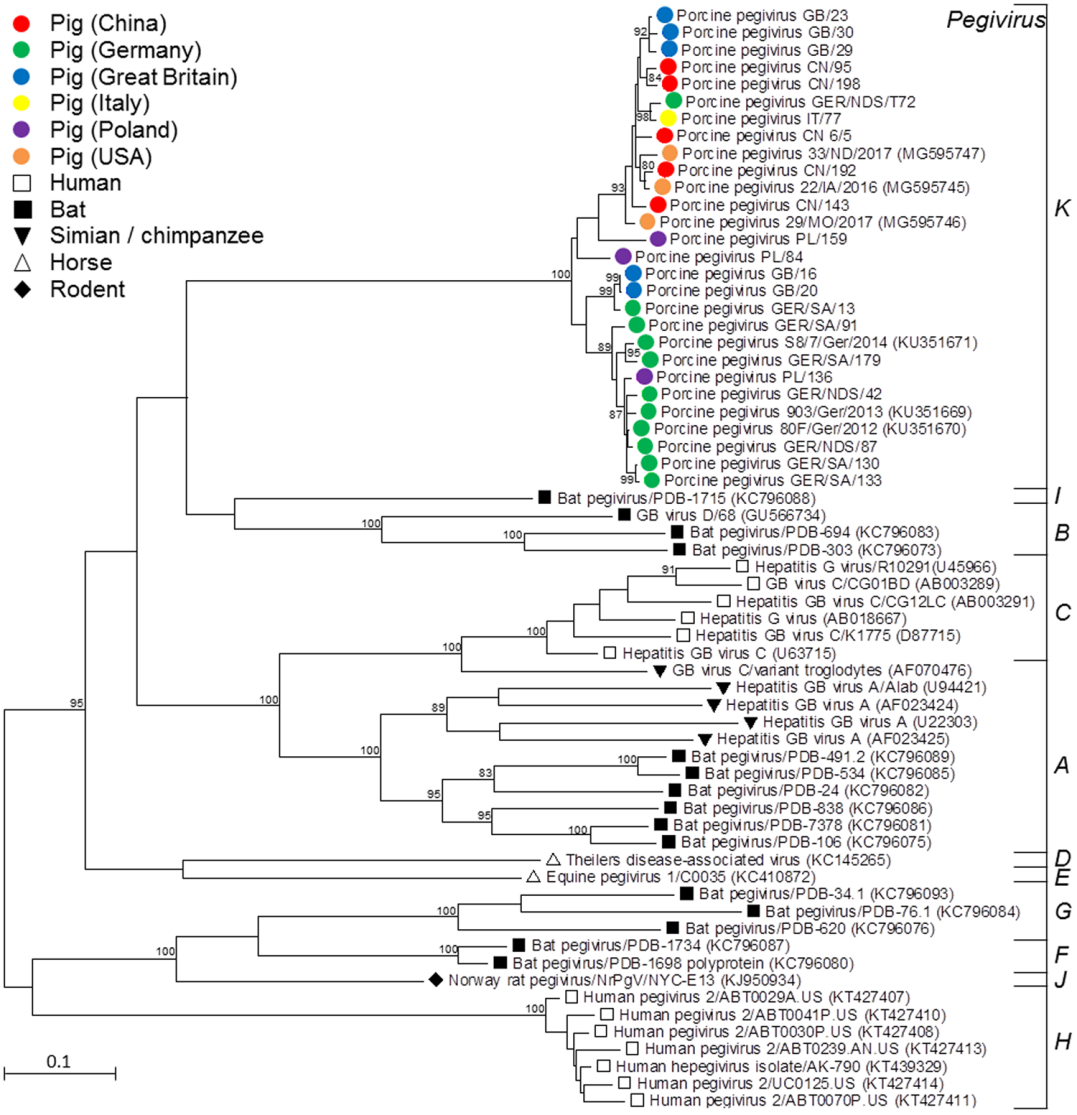
Phylogenetic analysis of porcine pegiviruses from different countries and other mammalian pegiviruses. Numbers along branches represent percentage bootstrap values (bootstrap values < 80% are not given). GenBank accession numbers are in parentheses. Scale bar indicates nucleotide substitutions per site. PPgV sequences are marked with a circle and the circle color indicates the country of origin. Pegivirus species A-K are indicated on the right.

Twenty-two distinct sequences shown here (Fig. 1) were submitted to GenBank. They displayed nucleotide sequence identities of >90%. According to phylogenetic analysis, PPgV formed a separate branch in the tree of pegiviruses and viral sequences segregated into two main clusters, one of which contained only sequences from Europe (Germany, Great Britain and Poland). Within the second main cluster, some branches contained sequences recovered from animals in Europe (GER/NDS/T72 and IT/77) in close proximity to variants from China (i.e. CN/6/5) and USA (i.e. 33/ND/2017)^24^.

Overall, the most closely related pegivirus sequence found in other species when compared to PPgV was bat pegivirus sequence PDB-1715 (GenBank KC796088), which had a nucleotide sequence identity of 58.1% with PPgV GB/30. A human pegivirus type 2 sequence, ABT0070P.US (GenBank KT427411) had the lowest nucleotide identity (47.1%) compared to PPgV sequences. When comparing PPgV sequences with pegivirus sequences originating from horses, nucleotide identities ranged from 53.7% to 55.7%. The sequence identities between PPgV and rodent pegivirus were around 54%, while the identities with simian pegiviruses ranged from 50.0% to 55.8%.

### PPgV RNA in tissue samples

In tissue samples of PPgV positive pigs, PPgV RNA was most abundant in the liver (Table 3). Liver samples of animals A and B contained 343.9 and 142.5 viral RNA copies/mg tissue, respectively, while 119.3 copies/mg tissue were found in the liver of animal C using qRT-PCR. Serum samples of these animals contained 2,051.1 copies/µl (animal A), 388.6 copies/µl (animal B) and 157.0 copies/µl (animal C). PBMCs were only available from animal A and contained 46 copies/µl whole blood used for isolation (Table 3).

**Table 3:** Porcine pegivirus RNA quantities and fluorescence *in situ* hybridization results in blood and different tissues from two domestic pigs from Germany^1^.

Blood component/tissue	Animal A		Animal B	
	PPgV RNA copies	FISH	PPgV RNA copies	FISH
Serum	2,051.1 /µl	n.d.	388.6 /µl	n.d.
PBMCs	46 /µl whole blood	**positive**	n.d.*	n.d.
Liver	343.9 /mg	**positive**	142.5 /mg	**positive**
Thymus	30.5 /mg	**positive**	28.9 /mg	**positive**
Spleen	25.5 /mg	negative	23.8 /mg	n.d.
Bone marrow	17.0 /mg	negative	2.9 /mg	n.d.
Tonsils	10.2 /mg	negative	0 /mg	n.d.
Pancreas	2.8 /mg	negative	7.7 /mg	n.d.
Mandibular lymph nodes	15.8 /mg	negative	0 /mg	n.d.
Pancreatic lymph nodes	n.d.	negative	n.d.	n.d.

^1^PPgV, porcine pegivirus; FISH, fluorescence *in situ* hybridization; n.d., not determined; boldface indicates positive FISH results; *fresh blood was not available.

**Figure 2:**
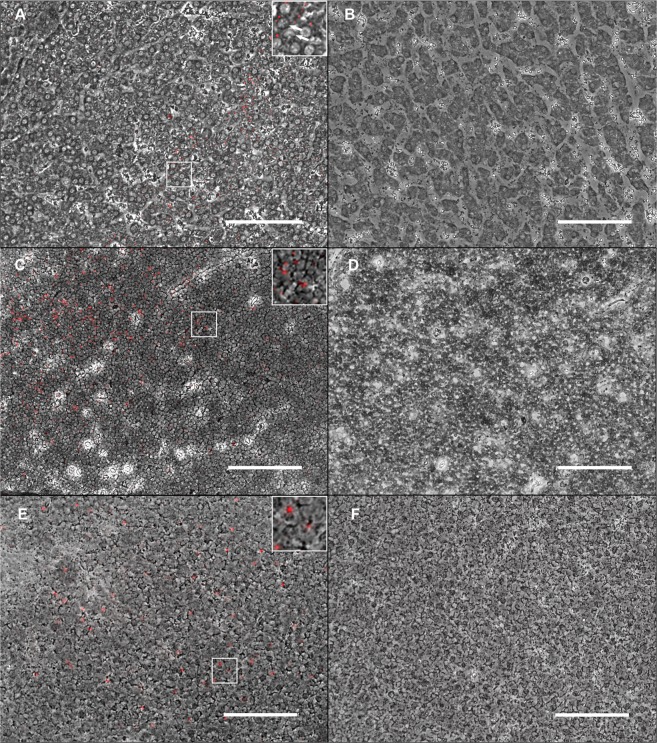
Fluorescence *in situ* hybridization of porcine pegivirus (PPgV) positive and negative pigs using a PPgV specific probe; overlay phase contrast and immunofluorescence; bar = 100 µm. (**A**) Single hepatocytes of the liver of a PPgV positive pig showed an intracytoplasmic positive signal for PPgV using a PPgV specific probe, also shown at higher magnification in the insert; arrows: nuclei of hepatocytes surrounded by intracytoplasmic, red, positive signals. (**B**) The liver of a PPgV negative pig lacked a PPgV specific signal. (**C**) Within the thymus of a PPgV positive pig, scattered cells showed an intracytoplasmic red positive signal for PPgV, also shown at higher magnification in the insert. (**D**) Within the thymus of a PPgV negative pig, all cells were negative for PPgV using a PPgV specific probe. (**E**) Several PBMCs from a PPgV positive pig showed a red positive signal using a PPgV specific probe, also shown at higher magnification in the insert. (**F**) PBMCs from a PPgV negative pig were negative for PPgV.

FISH was used to investigate the liver, thymus, PBMCs and different lymph nodes of animal A, as well as the liver and thymus of animal B, and respective tissues of negative control pigs. PPgV specific signals were detected in the liver of both PPgV positive pigs (Table 3; Fig. 2). Furthermore, several cells of the medullary and cortical region of the thymus of animals A and B were observed to be virus positive using the PPgV specific probe. Additionally, PBMCs of animal A were found to be virus positive in FISH, while lymph nodes, spleen, tonsils, bone marrow and pancreas of animal A tested virus negative. The non-probe incubation as well as the PPgV PCR-negative pigs showed no detectable positive area in the same tested organs, respectively. During necropsy of animal A, multifocal, mild, subendocardial hemorrhages were present. Histopathology showed a mild, portally accentuated, lymphohistiocytic hepatitis, a mild, diffuse infiltration of eosinophils within the thymus, tonsils and lymph nodes and single multinucleated giant cells within the medullary part of the thymus. Furthermore, lymph nodes revealed a mild, diffuse sinus histiocytosis. A moderate, focal, perivascular, lymphoplasmahistiocytic, partially eosinophilic dermatitis was present at the pinna. Additionally, a mild endocardiosis, a mild, lymphohistiocytic epicarditis and a mild to moderate, focal, follicular, lymphocytic conjunctivitis were observed.

## Discussion

The genus *Pegivirus* has grown in recent years, as new viruses were identified in different hosts. Yet little is known about their pathogenicity and the impact on the host’s immune response. In this study, our aim was to gain detailed insights into the distribution of PPgV in different parts of the world and the genetic diversity of PPgV. Viral RNA was detected in serum samples from domestic pigs from various European countries and China, with an overall individual detection rate of 2.7%.

Investigation of three different age groups from Europe and Asia showed a lower PPgV positive rate in younger animals such as piglets (1.9%) and fattening pigs (1.2%) than in adult animals (3.4%). This observation is concordant with the results from a recently published study from China; however, the increase in PPgV positive rate was more prominent there (1.6–11.3%). Focusing on samples from China, we found similar results: 1.0% detection rate in piglets and 9.7% detection rate in sows and boar^25^.

The PPgV positive rates found in this study differ between countries. While no samples were PPgV positive from Switzerland, Serbia and Taiwan, samples from Germany, Poland and Italy have a positive rate similar to the one described previously for German domestic pigs (2.2%)^9^. High detection rates in China (7.8%) and Great Britain (10.3%) found here are nonetheless lower than the positive rate observed in the USA (15.1%) in a previous study^24^. In humans, HPgV prevalence ranges from 0.5 to 5% in healthy blood donors from developed countries, but is higher in blood donors from developing countries (5–18.9%), and in individuals co-infected with blood borne or sexually transmitted diseases, like hepatits C virus or HIV-1^30–32^. Equine pegivirus (EPgV) has been found in 12 of 328 horses (3.7%) from Europe and 7 of 74 horses (9.5%) from USA^4,12^, thus showing similar detection rates as PPgV. The divergence in PPgV detection rates suggests uneven distribution of virus infection and local spread of PPgV. This may be caused by the occurrence of other infectious diseases in pig populations, similar to observations in humans with co-infection, and needs to be studied further. The viral loads determined here (2.4 × 10^2^ to 2 × 10^6^ copies/ml) are similar to EPgV RNA loads described in one study, which ranged from 3.2 × 10^4^ to 3.2 × 10^6^ RNA copies/ml^4^. However, another study found higher EPgV viral loads (4.1 × 10^5^ to 2.0 × 10^9^ RNA copies/ml)^12^, and the mean RNA load of HPgV in human plasma typically reaches >1 × 10^7^ copies/ml. This may suggest lower replication of PPgV *in vivo* compared to HPgV and EPgV.

Although HPgV does not appear to be hepatotropic, high amounts of PPgV RNA in the porcine liver shown by qRT-PCR and *in situ* techniques suggest that viral RNA may accumulate in the liver or even that PPgV has the ability to replicate in hepatocytes. However, this hypothesis will have to be investigated in future studies, as well as whether PPgV infections might be the cause of histopathological changes in the liver, as seen in animal A. Moreover, presence of PPgV RNA in PBMCs and in the thymus supports lymphotropism analogical to HPgV^22^. Positive FISH results in primary but not secondary lymphoid organs, such as spleen or lymph nodes, imply that the virus might replicate in the thymus and spread to other tissues (e.g. the liver) via PBMCs, but successfully evades recognition by the immune system, which could lead to a persistent infection in the host. Despite significant amounts of viral RNA detected in cells and tissues, highest viral loads were present in the serum of infected animals. With regard to this, low amounts of PPgV RNA in further organs and tissues can most probably be attributed to blood residues. Possible presence of viral RNA in serum microvesicles and associated virus uptake by PBMCs, as seen for HPgV, remain to be determined^22^.

Phylogenetic analyses showed close genetic relationships among PPgV sequences from different countries, like sequences GER/NDS/T72 and CN/6/5. This could suggest virus spread by international trade with pigs or pig products, such as feed. While all wild boar samples were tested negative for PPgV RNA in this study, other porcine viruses from the family *Flaviviridae*, such as APPV, have been shown to be present at a higher rate in wild boar (19%) than in domestic pigs from Germany (6.2%)^29,33^. For APPV, virus transmission between wild boar and domestic pigs appears likely, as strains originating from wild and domestic animals show genetic distance of as little as 6.6%^29^. However, due to the comparatively low prevalence of PPgV in Germany, transmission of the virus from domestic pigs to wild boar and vice versa may be limited. Only samples from wild boar hunted in northern Germany entered the present study. Future studies with extended sampling will reveal whether PPgV is also absent in wild boar from other geographical regions. As genome detection alone may result in underestimation of virus dissemination, upcoming investigations of samples from domestic pigs, wild boar and other species will also address serological reactions upon infection with PPgV.

These results manifest that PPgV, like other pegiviruses, is distributed over several continents. It can be hypothesized that putative immune modulatory effects of PPgV infections are implicated in pig health worldwide. Detection of PPgV RNA in lymphoid cells suggests that the virus has the potential to affect the immune system of pigs. First insights into the cell- and organ tropism of PPgV suggest that the virus may be hepatotropic and/or lymphotropic. Future studies will clarify the pathogenic potential and immune modulatory effects of this newly discovered, widely distributed virus.

## Methods

1,736 serum samples from domestic pigs from different countries in Europe (Germany, Great Britain, Poland, Switzerland, Italy and Serbia) and Asia (mainland China and Taiwan) originating from 132 different herds were analyzed in this study. For samples collected in Great Britain, the number of herds was unknown. Samples included 108 piglets up to four weeks old, 923 fattening pigs over four weeks old, 557 sows and boar, and 148 pigs of unknown age and use. Samples were taken between 2014 and 2018, originated from apparently healthy domestic pigs and were taken within the framework of national veterinary health management in concordance with national legal and ethical regulations. Residual volumes of these samples were provided for use in the current study, therefore no ethical approval was required for use of these samples. In addition, 800 serum samples from hunted wild boar from Lower Saxony, Germany, were included. 456 of these wild boar samples were collected during the hunting seasons of 2015/2016 and 2016/2017 and were used in a previous study investigating APPV prevalence^29^. 344 additional wild boar samples were collected during the hunting season of 2017/2018. Furthermore, blood and post-mortem tissue samples originated from apparently healthy PPgV positive pigs (n = 3, animals A, B, and C) from the Clinic for Swine, Small Ruminants, Forensic Medicine and Ambulatory Services (University of Veterinary Medicine, Hannover) and PPgV negative control pigs (n = 2). To rule out presence of co-infections with APPV and porcine reproductive and respiratory syndrome virus (PRRSV), PPgV positive animals were also tested using RT-PCR and found negative for both viruses (data not shown). One pig (animal A) was submitted to the Department of Pathology, University of Veterinary Medicine Hannover. A full necropsy was performed and samples of 40 different tissues were collected and stored at −80 °C or fixed in 10% neutral buffered formalin and embedded in paraffin wax. For histopathological examination, 3 µm thick sections were stained with hematoxylin and eosin. Different organ and tissue samples and a liver sample originated from two further PPgV positive pigs, animal B and animal C, respectively. Control samples for FISH were taken from PPgV negative pigs. PBMCs from animal A and one negative control animal were isolated from ~1 ml blood by density gradient centrifugation with Histopaque (Merck, Darmstadt, Germany). Euthanasia and sampling were approved by Lower Saxony’s official authorities (LAVES AZ 15A602 and 17A195) and were carried out in accordance with German legislation (TierSchVersV).

RNA was isolated from 140 µl of serum using the QIAmp Viral RNA Mini Kit (QIAGEN, Hilden, Germany) according to the manufacturer’s instructions. Isolation of RNA from preweighed tissue samples was achieved using the NucleoSpin RNA kit (Macherey-Nagel, Düren, Germany) or the RNeasy Mini Kit (QIAGEN) according to the manufacturer’s instructions and RNA samples were stored at −80 °C until testing. For PPgV genome quantification, a TaqMan based qRT-PCR targeting the highly conserved NS3 encoding region with primers PPgV/fwd/7 (5′-GTCTATGCTGGTCACGGA-3′), PPgV/rev/8 (5′-CACTCATCGCAAATGACCAC-3′) and probe PPgV/pro/11 (5′-[6FAM]-CCATTTCGCGAACCACTGATTCCA-[BHQ1]-3′) was developed and verified using samples that were PPgV positive in a SYBR Green qRT-PCR (QIAGEN) described in an earlier study^9^. For the new PCR assay, an *in vitro* transcribed RNA copy standard was developed using MEGAscript Kit (ThermoFisher Scientific, Germany) to allow for absolute quantification of genome copies. Real-time RT-PCR was performed using the Mx3005P QPCR System (Agilent Technologies, Santa Clara, USA) and the QuantiTect Probe RT-PCR Kit (QIAGEN) according to the manufacturer’s instructions on samples and RNA standard dilutions. Briefly, 12.5 µl RT-PCR master mix, 0.25 µl reverse transcriptase, 0.8 pmol of each primer and 0.2 pmol of the probe, 5.25 µl water and 5 µl sample RNA were used in each reaction of 25 µl with the following temperature profile: 50 °C for 30 minutes, 95 °C for 15 minutes and 40 cycles of 94 °C for 15 seconds and 60 °C for 1 minute. Serum samples were initially screened in pools containing three to ten individual samples; subsequently samples from positive pools were tested individually.

For phylogenetic analysis, amplicons corresponding to a partial NS3 coding sequence were generated by one of the following two methods: a) RT-PCR with SuperScript III One-Step RT-PCR System with Platinum TaqDNA Polymerase (Life technologies, Germany) with primers PPgV/fwd/G1 (5′-CACCGGGCTGTTTCTGCTA-3′) and PPgV/rev/G4 (5′-TTCCTTCCACACCAACCCAT-3′), or b) cDNA synthesis with SuperScript II Reverse Transcriptase (Invitrogen, Germany) using random hexamers followed by nested PCR with outer primers PPgV/fwd/G1 and PPgV/rev/G4, and inner primers PPgV/fwd/G3 (5′-CGGGCTGTTTCTGCTAGGT-3′) and PPgV/rev/G2 (5′-CACCAACCCATCGAGGATCA-3′) using *Taq* polymerase included in the Maxima Hot Start Green PCR Master Mix (2X) (ThermoFisher Scientific) and the following cycling parameters: 95 °C for 4 min, 40 cycles of 95 °C for 30 s, 52 °C for 30 s, 72 °C for 75 s, and 72 °C for 10 min. PCR products with an expected length of 1,290 (method a) and 1,278 (method b) were purified using the GeneJET PCR Purification Kit (ThermoFisher Scientific) according to the manufacturer’s instructions and submitted to Sanger sequencing (FlexiRun, LGC Genomics, Germany) with primers PPgV/fwd/G3 and PPgV/rev/G2. Sequences were trimmed to a final length of 1041 base pairs and a multiple sequence alignment was performed with ClustalW implemented in BioEdit 7.0^34^. Phylogenetic trees were calculated in MEGA7 using the Maximum-likelihood method and the Kimura 2-parameter substitution model^35^ with 500 replicates for statistical evaluation.

FISH was performed on formalin-fixed, paraffin-embedded organ sections of two qRT-PCR positive pigs (animal A and B) and on the PBMC pellet of one pig (animal A) using a PPgV specific RNA probe covering parts of the PPgV NS3. The probe set (ViewRNA TYPE 1 Probe Set, ThermoFisher Scientific) covered positions 2–816 of a target sequence with 1172 nucleotides that overlapped with the partial PPgV sequence of animal A (GenBank MH979651). The procedure was carried out according to the manufacturer´s protocol with minor variations as previously described (ViewRNA TYPE 1 Probe Set; ViewRNA™ ISH Tissue Assay Kit (1-plex) and ViewRNA Chromogenic Signal Amplification Kit; ThermoFisher Scientific;)^36^. Briefly, sections were deparaffinized, boiled in pretreatment solution® at 90 °C for 10 minutes, digested by a protease QF® at 40 °C for 10 minutes and afterwards fixed. Hybridization to the specific probe was performed for 6 hours. Following preamplification and amplification steps, sections were stained with Fast Red Substrate and counterstained with Mayer´s hemalum® (Carl Roth GmbH, Karlsruhe, Germany). Images were acquired with an inverted fluorescence microscope (Olympus IX-70; Olympus Life Science Europe GmbH, Hamburg, Deutschland). The specificity of the probe was confirmed by including a non-probe incubation which served as system negative control and organ sections and cells of PPgV RT-PCR-negative pigs, respectively.

### Accession codes

The obtained DNA sequences were deposited in GenBank (accession numbers: MH979651-MH979672).
